# Conjunctival structure of glaucomatous eyes treated with anti-glaucoma eye drops: a cross-sectional study using anterior segment optical coherence tomography

**DOI:** 10.1186/s12886-020-01518-6

**Published:** 2020-06-19

**Authors:** Makoto Gozawa, Yoshihiro Takamura, Kentaro Iwasaki, Shogo Arimura, Masaru Inatani

**Affiliations:** Department of Ophthalmology, Faculty of Medical Sciences, University of Fukui, 23-3 Shimoaizuki, Matsuoka, Eiheiji, Yoshida, Fukui, 910-1193 Japan

**Keywords:** Glaucoma, Conjunctiva, Anterior segment optical coherence tomography, Anti-glaucoma eye drops

## Abstract

**Background:**

To determine the effect of various factors to the preservation rate of the conjunctival layer borderlines of glaucomatous eyes treated with anti-glaucoma eye drops.

**Methods:**

Anterior segment optical coherence tomography (AS-OCT) images of the bulbar conjunctiva of 328 eyes were analyzed with and without anti-glaucoma eye drops to quantify the preservation rates of the conjunctival layer borderlines.

**Results:**

More anti-glaucoma eye drops and a longer duration of administration were associated with lower preservation rates of the borderlines between both the conjunctival stroma/Tenon’s capsule (*P* < 0.001 and *P* < 0.001, respectively) and Tenon’s capsule/sclera (*P* < 0.001 and *P* < 0.001, respectively). Prostaglandin analogs and fixed combinations of β-blockers/prostaglandin analogs were prognostic factors for lower preservation rates of the borderlines between both the conjunctival stroma/Tenon’s capsule (*P* < 0.001 and *P* = 0.009, respectively) and Tenon’s capsule/sclera (*P* < 0.001 and *P* = 0.008, respectively).

**Conclusions:**

Numerous anti-glaucoma eye drops and their long-term administration are associated with the disruption of the bulbar conjunctival borderlines detected by AS-OCT.

## Background

Large clinical trials have revealed that reduction of intraocular pressure (IOP) in patients with glaucoma prevents the progression of glaucomatous optic neuropathy and visual field defects [1–3]. In the past decade, various anti-glaucoma eye drops have been developed, such as prostaglandin analogs, β-blockers, α-adrenergic-receptor agonists, carbonic anhydrase inhibitors (CAIs), Rho kinase inhibitors, and fixed combinations including β-blockers/CAIs and ß-blockers/prostaglandin analogs. Most patients with glaucoma are treated with various anti-glaucoma eye drops to maintain IOP reduction.

In most cases, long-term administration of anti-glaucoma eye drops is necessary for efficient IOP control. Although most anti-glaucoma eye drops do not cause severe systemic effects, some studies have demonstrated that long-term administration of anti-glaucoma eye drops induces histopathological and inflammatory changes in the conjunctival tissues. These changes are deleterious because altered histology of conjunctival tissue was shown to be a risk factor for surgical failure in patients who underwent trabeculectomy [4–12].

Anterior segment optical coherence tomography (AS-OCT) has facilitated the non-invasive imaging of conjunctival structures [13]. We previously demonstrated that AS-OCT images can detect conjunctival inflammatory responses and subconjunctival scarring after phacoemulsification and pars plana vitrectomy [14, 15]. However, no studies using AS-OCT have reported on the conjunctival structure after long-term administration of anti-glaucoma eye drops. Therefore, our study aimed to determine whether the structural features of conjunctival tissues after long-term administration of anti-glaucoma eye drops can be quantified using the AS-OCT images.

## Methods

### Patient selection

This study adhered to the tenets of the Declaration of Helsinki. We included patients with and without glaucoma who visited the University of Fukui Hospital between June 20, 2016 and February 23, 2018. We enrolled 199 patients (328 eyes) in this study using the following inclusion criteria: age ≥ 20 years and patients who had never been or had been treated with single or multiple anti-glaucoma eye drops for at least 1 month. We excluded patients with a history of any intraocular surgery or ocular trauma, those with dry eye diseases, or those using any type of drugs except anti-glaucoma drops. If both the patient’s eyes satisfied the inclusion criteria, both eyes were included in the study. Out of 26 secondary eyes, 16 eyes were exfoliation glaucoma. There are no previous reports about the disturbed structure in the conjunctival tissue of exfoliation glaucoma. Out of the remaining 10 eyes, 7 eyes are uveitic glaucoma with open-angle. However, the patients were not associated with active intraocular inflammation when AS-OCT were evaluated. Therefore, the eyes were not treated with the eye drops of corticosteroid. It seems to be less likely that ocular inflammation affected the status of conjunctiva.

### AS-OCT imaging and image analysis

We obtained AS-OCT images of the bulbar conjunctiva using the CASIA SS-1000 (Tomey, Nagoya, Japan). Superior bulbar conjunctiva was chosen because it is a common site for trabeculectomy. To obtain scanned OCT images of the superior regions of the bulbar conjunctiva, the patients gazed downward while keeping their head in a straight direction. On the image from iris view point, we chose the 12 o’-clock region of the bulbar conjunctiva as the superior conjunctiva. At the superior conjunctiva, we defined the intersection of the perpendicular line from the angle with the ocular surface as the corneal limbus on the cross-sectional image. Then, we defined the point 3 mm posterior to the limbus as the distal point measured. Then, we identified three conjunctival layers, i.e., the conjunctival epithelium, the conjunctival stroma, and Tenon’s capsule, as previously described [13]. We defined the conjunctival epithelium as a continuous, narrow, and low reflective surface layer. Beneath the conjunctival epithelium, the conjunctival stroma was defined as a highly reflective layer that is separated from the underlying low reflective layer (Tenon’s capsule). We measured the lengths of the borderlines among these conjunctival layers and the sclera by manually tracing each borderline using the central corneal thickness measurement software included in the OCT device. To quantify the disruption of the bulbar conjunctival borderlines, we defined the preservation rate as the length of the borderline divided by 3 mm × 100% as previously reported [14]. One technician who was blinded to the aim of the study processed and analyzed all the images.

### Statistical analyses

We consulted with a professional statistician (Department of Medical Statistics, Satista Co., Ltd., Kyoto, Japan). We conducted general linear mixed model (GLMM) analyses to evaluate the effects of the eye drops on the preservation rates after adjusting within-subjects factor due to the inclusion of both eyes data. First, to extract the background characteristics as the confounding factors, we performed a univariable and multivariable GLMM analysis with the preservation rates as the dependent variables, the background characteristics as the fixed factor and the subjects as the random factor (multiple analysis 1). Variables with collinearity were excluded in this analysis. The collinearity was evaluated by calculating variance inflation factor (VIF), and VIF = 10 was used as a threshold. Factors with *P* < 0.05 in this analysis were considered potential confounding factors. Next, to correct for confounding factors, we performed a multiple GLMM analysis with the preservation rates as the dependent variables and with each type of anti-glaucoma eye drops and the confounding factors as the fixed factor and subjects as random factor (multiple analysis 2). In addition, we used a stepwise method for variable selection to create a model for simultaneous analysis of anti-glaucoma eye drops. (multiple analysis 3). A *P*-value of < 0.05 was considered statistically significant. We performed all statistical analyses using SPSS v.23.0 software for Windows (IBM Japan, Tokyo, Japan).

## Results

### Patient characteristics

Table 1 summarizes the baseline characteristics, Supplemental file 1 summarizes the types of anti-glaucoma eye drops, and supplemental file 2 presents the types of combination therapy used for the included patients. We counted the number of fixed combinations of β-blockers/CAIs and β-blockers/prostaglandin analogs as one bottle, respectively.

**Table 1:** Patient characteristics

Characteristics	n (Patients)	n (Eyes)	Mean ± SD	Range
Sex				
Male	83	131		
Female	116	197		
Age (years)			72.1 ± 12.3	18–96
Number of anti-glaucoma eye drops			0.79 ± 1.25	0–4
Duration of administration (months)			7.5 ± 14.9	0–66
No glaucoma without anti-glaucoma drops	138	236		
Glaucoma without anti-glaucoma drops	1	1		
Glaucoma with anti-glaucoma drops	60	101		
Type of glaucoma				
Primary open-angle glaucoma	30	52		
Primary angle closure glaucoma	3	6		
Normal-tension glaucoma	11	17		
Exfoliation glaucoma	12	16		
Neovascular glaucoma	2	3		
Other secondary glaucomas	6	7		

### AS-OCT images

**Fig. 1:**
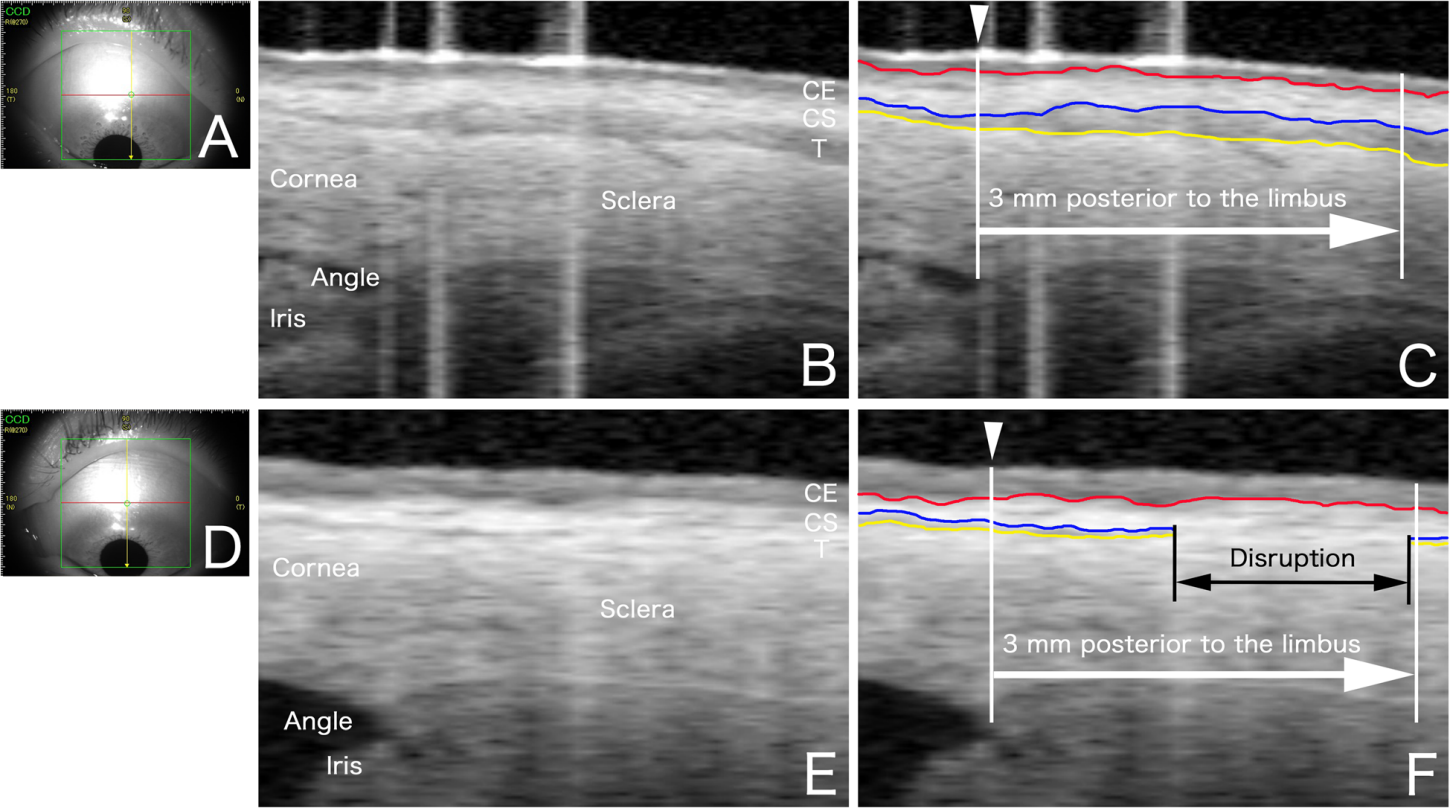
Images of the bulbar conjunctiva in a normal subject and a patient using multiple glaucoma eye drops. **a**, **d** Image from iris viewpoint. The anterior segment scan (solid yellow line) was performed at the superior conjunctiva. White arrowheads in C and F indicates the corneal limbus defined by the intersection of the perpendicular line from the angle with the ocular surface. **a-c** Images obtained from a patient without anti-glaucoma eye drops (65-year-old man). A continuous, narrow, low reflectivity surface layer (conjunctival epithelium [CE]) was present. Below the conjunctival epithelial layer, a highly reflective layer (conjunctival stroma [CS]) was separated from the underlying low reflectivity layer (Tenon’s capsule [T]). The sclera can be observed as a highly reflective region beneath Tenon’s capsule. Red, blue, and yellow lines indicate the borderlines of the conjunctival epithelium/stroma, stroma/Tenon’s capsule, and Tenon’s capsule/sclera, respectively. **d-f** Images obtained from a glaucoma patient (63-year-old man) with multiple anti-glaucoma eye drops (prostaglandin analog + a combination of β-blocker/CAI). The disruption of the bulbar conjunctival borderlines of both the stroma/Tenon’s capsule and Tenon’s capsule/sclera was observed, but the borderlines of the conjunctival epithelium/conjunctival stroma were preserved

Figure 1 displays the AS-OCT images of the superior bulbar conjunctiva in an eye without anti-glaucoma eye drops and in an eye that was treated with multiple glaucoma eye drops. The AS-OCT could detect the three-layered structure in the conjunctiva of eyes without anti-glaucoma eye drops. In the eye with multiple anti-glaucoma eye drops (prostaglandin analog + the fixed combination of β-blocker/CAI), the borderlines of the conjunctival layers were obscure, except for the conjunctival epithelium/stroma.

### Preservation rates of the conjunctival layer borderlines

Table 2a and supplemental file 3a shows that the preservation rates significantly decreased with aging in multiple analysis 1 (β = − 0.114, *P* = 0.038). We found no significant differences among the uses of different anti-glaucoma eye drops in the univariate analysis and in multiple analysis 2 (Table 2b and supplemental file 3b).

**Table 2:** The univariate and general linear mixed model (GLMM) analyses on the conjunctival epithelium/conjunctival stroma preservation rate

a. The effects of background characteristics					
	Univariable analysis		Multiple analysis 1		
	β	*p*-value	β	*p*-value	VIF
Sex (male vs. female)	−0.051	0.281	−0.058	0.234	1.08
Age (years)	−0.114	**0.033**	−0.114	**0.038**	1.06
Number of anti-glaucoma eye drops	−0.045	0.326	−0.089	0.132	1.74
Duration of administration (months)	0.015	0.768	0.078	0.227	1.72
b. The effects of anti-glaucoma eye drops					
	Univariable analysis		Multiple analysis 2		
	β	*p*-value	β	*p*-value	
Prostaglandin analogs	−0.046	0.331	− 0.047	0.299	
α2-receptor agonist	−0.065	0.160	−0.064	0.161	
Rho kinase inhibitor	0.031	0.440	0.028	0.488	
The fixed combination of β-blockers/CAIs	−0.025	0.564	−0.024	0.579	
The fixed combination of β-blockers/prostaglandin analogs	−0.052	0.303	−0.050	0.313	
β-blockers	0.012	0.821	0.006	0.909	
CAIs	−0.038	0.449	−0.048	0.333	

Multiple analysis 1, multiple GLMM analysis with the preservation rate and with background characteristics; Multiple analysis 2, multiple GLMM analysis with the preservation rate and with the eye drops after adjusted for confounding factor (age)

*CAI* carbonic anhydrase inhibitors, *VIF* variance inflation factor

Table 3a and supplemental file 4a shows that more eye drops (β = − 0.454, *P* < 0.001) and a longer duration of administration (β = − 0.349, *P* < 0.001) were associated with lower preservation rates in multiple analysis 1. In Table 3b and supplemental file 4b, multiple analysis 3 shows that prostaglandin analogs (β = − 0.183, *P* < 0.001) and the fixed combinations of β-blockers/prostaglandin analogs (β = − 0.091, *P* = 0.009) are prognostic factors for lower preservation rates; however, α2-receptor agonist (β = 0.103, *P* = 0.021) was associated with a significantly higher preservation rate than the other types of eye drop.

**Table 3:** The univariate and general linear mixed model (GLMM) analyses on the conjunctival stroma/Tenon’s capsule preservation rate

a. The effects of background characteristics							
	Univariable analysis		Multiple analysis 1				
	β	*p*-value	β	*p*-value	VIF		
Sex (male vs. female)	−0.010	0.867	−0.027	0.377	1.08		
Age (years)	−0.008	0.874	0.015	0.593	1.06		
Number of anti-glaucoma eye drops	−0.654	**< 0.001**	− 0.454	**< 0.001**	1.74		
Duration of administration (months)	−0.660	**< 0.001**	−0.349	**< 0.001**	1.72		
b. The effects of anti-glaucoma eye drops							
	Univariable analysis		Multiple analysis 2		Multiple analysis 3		
	β	*p*-value	β	*p*-value	β	*p*-value	VIF
Prostaglandin analogs	−0.586	**< 0.001**	−0.174	**0.001**	**−0.183**	**< 0.001**	1.72
α2-receptor agonist	−0.388	**< 0.001**	0.142	**0.001**	**0.103**	**0.021**	1.57
Rho kinase inhibitor	−0.150	**0.001**	0.007	0.824			
The fixed combination of β-blockers/CAIs	−0.439	**< 0.001**	− 0.047	0.251			
The fixed combination of β-blockers/prostaglandin analogs	−0.337	**< 0.001**	− 0.055	0.117	**−0.091**	**0.009**	1.33
β-blockers	−0.240	**< 0.001**	−0.039	0.295			
CAIs	−0.243	**< 0.001**	0.083	**0.019**			

Multiple analysis 1, multiple GLMM analysis with the thickness of the preservation rate and with background characteristics; Multiple analysis 2, multiple GLMM analysis with the preservation rate and with the eye drops after adjusted for confounding factor (number of anti-glaucoma eye drops and duration of administration).; Multiple analysis 3, the stepwise method for variable selection

*CAI* carbonic anhydrase inhibitors, *VIF* variance inflation factor

Table 4a and supplemental file 5a shows that in multiple analysis 1, more anti-glaucoma eye drops (β = − 0.408, *P* < 0.001) and a longer duration of administering anti-glaucoma eye drops (β = − 0.374, *P* < 0.001) were associated with lower preservation rates. In Table 4b and supplemental file 5b, multiple analysis 3 shows that prostaglandin analogs (β = − 0.191, *P* < 001) and the fixed combinations of β-blockers/prostaglandin analogs (β = − 0.091, *P* = 0.008) were prognostic factors for lower preservation rates; however, α2-receptor agonist (β = 0.146, *P* = 0.001) was associated with a significantly higher preservation rate than the other types of eye drop.

**Table 4:** The univariate and general linear mixed model (GLMM) analyses on the Tenon’s capsule/sclera preservation rate

a. The effects of background characteristics							
	Univariable analysis		Multiple analysis 1				
	β	*p*-value	Β	*p*-value	VIF		
Sex (male vs. female)	−0.008	0.886	− 0.027	0.384	1.08		
Age (years)	−0.004	0.941	0.027	0.354	1.06		
Number of anti-glaucoma eye drops	−0.624	**< 0.001**	− 0.408	**< 0.001**	1.74		
Duration of administration (months)	−0.653	**< 0.001**	−0.374	**< 0.001**	1.72		
b. The effects of anti-glaucoma eye drops							
	Univariable analysis		Multiple analysis 2		Multiple analysis 3		
	β	*p*-value	β	*p*-value	β	*p*-value	VIF
Prostaglandin analogs	−0.569	**< 0.001**	−0.193	**< 0.001**	**−0.191**	**< 0.001**	1.72
α2-receptor agonist	−0.352	**< 0.001**	0.186	**< 0.001**	**0.146**	**0.001**	1.57
Rho kinase inhibitor	−0.196	**< 0.001**	− 0.045	0.147			
The fixed combination of β-blockers/CAIs	−0.435	**< 0.001**	−0.069	0.102			
The fixed combination of β-blockers/prostaglandin analogs	−0.321	**< 0.001**	− 0.053	0.136	**−0.091**	**0.008**	1.33
β-blockers	−0.149	**0.010**	−0.004	0.907			
CAIs	−0.234	**< 0.001**	0.070	0.052			

Multiple analysis 1, multiple GLMM analysis with the thickness of the preservation rate and with background characteristics; Multiple analysis 2, multiple GLMM analysis with the preservation rate and with the eye drops after adjusted for confounding factor (number of anti-glaucoma eye drops and duration of administration).; Multiple analysis 3, the stepwise method for variable selection

*CAI* carbonic anhydrase inhibitors, *VIF* variance inflation factor

## Discussion

In vitro and ex vivo studies have demonstrated that long-term administration of anti-glaucoma eye drops induces significant histopathological and inflammatory changes in conjunctival tissues [4–12]. However, these findings have used human or animal histological specimens that did not provide in vivo measurements of the conjunctival structures. Our study is unique because the structural features of conjunctiva caused by long-term administration of anti-glaucoma eye drops were non-invasively quantified by AS-OCT.

This study identified the use of prostaglandin analogs and the fixed combinations of β-blocker/prostaglandin analog eye drops as prognostic factors for decreasing the preservation rate of the borderlines between the conjunctival stroma/Tenon’s capsule and Tenon’s capsule/sclera, suggesting that prostaglandin analogs are the main prognostic factor for lower preservation rates. Prostaglandin analogs are currently considered the first line of administration for glaucoma patients because of their efficacy, systemic tolerability, and high patient adherence to the once-daily treatment. However, prostaglandin analogs provoke a conjunctival inflammatory reaction because human-leukocyte-associated antigen (HLA)-DR is expressed in the conjunctiva even after a short period of administration, independent of the types of prostaglandin analogs used [16]. Preservative-free latanoprost also promotes the activation of P38-NF-κB signaling and upregulates the expressions of cytokines, followed by CD4+ T cell infiltration in the mouse conjunctiva [17]. Matrix metalloproteinases (MMPs) are upregulated, but tissue inhibitors of metalloproteinase (TIMPs) are downregulated in rat conjunctival tissue when prostaglandin analogs are administered, suggesting that prostaglandin analogs may enhance extracellular matrix degradation [18]. An experiment on the effects of prostaglandin analogs on human conjunctiva showed that the altered expressions of MMP-3 and TIMP-2 from the fibroblasts resulted in the remodeling of the extracellular matrix [19]. Moreover, human conjunctiva treated with prostaglandin analogs contains amorphous material [20]. These data indicate that the lower preservation rate of the borderlines between the conjunctival stroma/Tenon’s capsule and Tenon’s capsule/sclera in eyes treated with prostaglandin analogs or fixed combinations of β-blockers/prostaglandin analogs may reflect the remodeling of extracellular matrices in the conjunctiva due to prostaglandin analog-induced inflammation.

Interestingly, the α2-receptor agonist was significantly associated with higher preservation rates of the borderlines than other types of eye drops. In rat conjunctiva treated with brimonidine, the concentrations of inflammatory cytokines, such as IL-1β, IL-2, and IL-6, were significantly lower than rates in the control conjunctiva [21]. Additionally, there were significantly fewer inflammatory cells in the conjunctiva treated with brimonidine than in conjunctiva treated with timolol or latanoprost [22]. The reduced inflammation in the conjunctiva may contribute to the higher preservation rates of the borderlines of the conjunctival stroma/Tenon’s capsule and Tenon’s capsule/sclera in the α2-receptor agonist.

Preservatives present in eye drops suppress microbial activity and preserve the sterility of ophthalmic formulations for multidose drug bottles. However, long-term administration of anti-glaucoma eye drops with preservatives induces significant histopathological and inflammatory changes in the conjunctival tissues, such as increased numbers of fibroblasts and inflammatory cells. These conditions induce postoperative fibrotic responses in the conjunctival tissues, resulting in bleb failure and a reduced success rate of filtration surgery. The inflammation induced by preservatives may reduce the preservation rates of the borderlines between the conjunctival stroma/Tenon’s capsule and Tenon’s capsule/sclera. Interestingly, Purite®, the preservative in brimonidine, is known to cause less lymphocytic infiltration in the conjunctival stroma than other preservatives [22]. The reduced lymphocytic infiltration in the conjunctiva due to Purite® may contribute to the higher preservation rates of the borderlines of conjunctival stroma/Tenon’s capsule and Tenon’s capsule/sclera.

The present study has some limitations. First, although the AS-OCT images quantified the disruption of the bulbar conjunctival borderlines, the associations between the observations detected by the AS-OCT images and the surgical outcomes of trabeculectomy remain unknown. Before trabeculectomy, AS-OCT images of the conjunctiva should be taken in eyes that were treated with various anti-glaucoma eye drops to identify the relationship. Second, the present study was unable to determine whether the disruption of the bulbar conjunctival borderlines is reversible because the study design was cross-sectional. If the disruption is reversible, the cessation of anti-glaucoma eye drops would be beneficial for eyes that will be treated with a trabeculectomy. Third, there was no histological confirmation of what the disruption of conjunctival layer borderlines caused by long-term administration of anti-glaucoma eye drops represents. A longitudinal study is required for further information.

## Conclusion

In conclusion, AS-OCT images display the disruption of the bulbar conjunctival borderlines in eyes that have experienced long-term administration of anti-glaucoma drops. Assessments of the conjunctiva based on AS-OCT images may contribute to better therapeutic management for glaucoma patients treated with anti-glaucoma eye drops.

## Supplementary information

**Additional file 1: Supplemental file 1.** Type of anti-glaucoma eye drops.

**Additional file 2: Supplemental file 2.** The combination of anti-glaucoma eye drops.

**Additional file 3: Supplemental file 3.** The univariate and general linear mixed model (GLMM) analyses on the conjunctival epithelium/conjunctival stroma preservation rate. a. The effects of background characteristics. b. The effects of anti-glaucoma eye drops.

**Additional file 4: Supplemental file 4.** The univariate and general linear mixed model (GLMM) analyses on the conjunctival stroma/Tenon’s capsule preservation rate. a. The effects of background characteristics. b. The effects of anti-glaucoma eye drops.

**Additional file 5: Supplemental file 5.** The univariate and general linear mixed model (GLMM) analyses on the Tenon’s capsule/sclera preservation rate. a. The effects of background characteristics. b. The effects of anti-glaucoma eye drops.

## Data Availability

The research article data used to support the findings of this study are included within the article and are available from the corresponding author upon request.

## Abbreviations

AS-OCT: Anterior segment optical coherence tomography
IOP: Intraocular pressure
CAIs: Carbonic anhydrase inhibitors
GLMM: General linear mixed model
VIF: Variance inflation factor
HLA: Human-leukocyte-associated antigen
MMPs: Matrix metalloproteinases
TIMPs: Tissue inhibitors of metalloproteinase
