# Supplementary material for: Conjunctival structure of glaucomatous eyes treated with anti-glaucoma eye drops: a cross-sectional study using anterior segment optical coherence tomography
Source: BMC Ophthalmol. 2020 Jun 19;20:244. doi: 10.1186/s12886-020-01518-6 (PMC7304144; doi:10.1186/s12886-020-01518-6)
Supplement: Supplementary file 1 — Additional file 1: Supplemental file 1. Type of anti-glaucoma eye drops. [file 12886_2020_1518_MOESM1_ESM.docx]

Supplemental file 1. Type of anti-glaucoma eye drops

| Class | Generic name / trade name | Preservative | n (Patients) | Total n (Patients) | n (Eyes) | Total n (Eyes) |
| --- | --- | --- | --- | --- | --- | --- |
| Prostaglandin analogs | Latanoprost/Xalatan | BAK | 20 | 49 | 27 | 79 |
|  | Tafluprost/Tapros | BAK | 7 |  | 13 |  |
|  | Bimatoprost/Lumigan | BAK | 16 |  | 29 |  |
|  | Travoprost/Travatan Z | SofZia | 6 |  | 10 |  |
| α2-receptor agonist | Brimonidine tartrate/Aiphagan | Purite | 31 | 31 | 48 | 48 |
| Rho kinase inhibitor | Ripasudil/Glanatec | BAK | 10 | 10 | 15 | 15 |
| The fixed combinations of β-blockers/CAIs | Dorzolamide/timolol maleate/Cosopt | BAK | 28 | 39 | 46 | 63 |
|  | Brinzolamide/timolol maleate/Azorga | BAK | 11 |  | 17 |  |
| The fixed combinations of β-blockers/prostaglandin analogs | Latanoprost/timolol/Xalcom | BAK | 5 | 12 | 7 | 16 |
|  | Tafluprost/timolol maleate/Tapcom | BAK | 4 |  | 4 |  |
|  | Travoprost/timolol/DuoTrav | BAK | 3 |  | 5 |  |
| β-blockers | Timolol maleate/Timoptol-XE | BDD | 3 | 4 | 5 | 6 |
|  | Carteolol/Mikelan | BAK | 1 |  | 1 |  |
| CAIs | Brinzolamide/Azopt | BAK | 11 | 13 | 14 | 18 |
|  | Dorzolamide/Trusopt | BAK | 2 |  | 4 |  |

β-blocker, β-adrenergic-receptor antagonists; CAIs, carbonic anhydrase inhibitors; SD, standard deviation; BAK, benzalkonium chloride; BDD, benzododecinium bromide
