# Supplementary material for: Conjunctival structure of glaucomatous eyes treated with anti-glaucoma eye drops: a cross-sectional study using anterior segment optical coherence tomography
Source: BMC Ophthalmol. 2020 Jun 19;20:244. doi: 10.1186/s12886-020-01518-6 (PMC7304144; doi:10.1186/s12886-020-01518-6)
Supplement: Supplementary file 2 — Additional file 2: Supplemental file 2. The combination of anti-glaucoma eye drops. [file 12886_2020_1518_MOESM2_ESM.docx]

Supplemental file 2. The combination of anti-glaucoma eye drops

| Type of combination (generic name/trade name) | n (Patients) | n (Eyes) |
| --- | --- | --- |
| Latanoprost/Xalatan | 3 | 4 |
| Latanoprost/Xalatan + Timolol maleate/Timoptol-XE | 1 | 1 |
| Latanoprost/Xalatan + Timolol maleate/Timoptol-XE + Brinzolamide/Azopt | 1 | 2 |
| Latanoprost/Xalatan + Brimonidine tartrate/Aiphagan | 2 | 3 |
| Latanoprost/Xalatan + Brimonidine tartrate/Aiphagan + Brinzolamide/Azopt | 1 | 1 |
| Latanoprost/Xalatan + Brimonidine tartrate/Aiphagan + Brinzolamide/timolol maleate/Azorga | 2 | 2 |
| Latanoprost/Xalatan + Brimonidine tartrate/Aiphagan + Brinzolamide/timolol maleate/Azorga + Ripasudil/Glanatec | 2 | 2 |
| Latanoprost/Xalatan + Dorzolamide/timolol maleate/Cosopt | 3 | 3 |
| Latanoprost/Xalatan + Brinzolamide/timolol maleate/Azorga | 1 | 1 |
| Latanoprost/Xalatan + Brinzolamide/timolol maleate/Azorga + Ripasudil/Glanatec | 2 | 3 |
| Latanoprost/Xalatan + Brimonidine tartrate/AIphagan + Dorzolamide/timolol maleate/Cosopt | 4 | 5 |
| Bimatoprost/Lumigan + Dorzolamide/timolol maleate/Cosopt | 3 | 5 |
| Bimatoprost/Lumigan + Brinzolamide/timolol maleate/Azorga | 1 | 2 |
| Bimatoprost/Lumigan + Ripasudil/Glanatec + Brinzolamide/timolol maleate/Azorga | 3 | 4 |
| Bimatoprost/Lumigan + Brimonidine tartrate/Aiphagan | 1 | 2 |
| Bimatoprost/Lumigan + Brimonidine tartrate/Aiphagan + Dorzolamide/timolol maleate/Cosopt | 8 | 15 |
| Bimatoprost/Lumigan + Brimonidine tartrate/Aiphagan + Brinzolamide/timolol maleate/Azorga | 1 | 2 |
| Bimatoprost/Lumigan + Brimonidine tartrate/Aiphagan + Dorzolamide/timolol maleate/Cosopt + Ripasudil/Glanatec | 1 | 1 |
| Brimonidine tartrate/Aiphagan + Latanoprost/timolol/Xalcom + Brinzolamide/Azopt | 3 | 3 |
| Brimonidine tartrate/Aiphagan + Latanoprost/timolol/Xalcom + Dorzolamide/Trusopt | 1 | 2 |
| Brimonidine tartrate/Aiphagan + Tafluprost/timolol maleate/Tapcom + Brinzolamide/Azopt | 1 | 1 |
| Brimonidine tartrate/Aiphagan + Latanoprost/timolol/Xalcom + Ripasudil/Glanatec | 1 | 2 |
| Brimonidine tartrate/Aiphagan + Travoprost/timolol/DuoTrav + Brinzolamide/Azopt | 1 | 2 |
| Tafluprost/Tapros | 2 | 3 |
| Tafluprost/Tapros + Dorzolamide/timolol maleate/Cosopt | 1 | 2 |
| Tafluprost/Tapros + Brimonidine tartrate/Aiphagan + Dorzolamide/timolol maleate/Cosopt | 1 | 2 |
| Tafluprost/Tapros + Brimonidine tartrate/Aiphagan + Timolol maleate/Timoptol-XE + Brinzolamide/Azopt | 1 | 2 |
| Tafluprost/Tapros + Dorzolamide/timolol maleate/Cosopt | 1 | 2 |
| Tafluprost/Tapros + Dorzolamide/timolol maleate/Cosopt + Ripasudil/Glanatec | 1 | 2 |
| Travoprost/Travatan Z | 2 | 2 |
| Travoprost/Travatan Z + Dorzolamide/timolol maleate/Cosopt | 4 | 5 |
| Travoprost/Travatan Z + Brimonidine tartrate/Aiphagan + Dorzolamide/timolol maleate/Cosopt | 2 | 3 |
| Dorzolamide/timolol maleate/Cosopt | 2 | 3 |
| Brinzolamide/timolol maleate/Azorga | 1 | 1 |
| Tafluprost/timolol maleate/Tapcom | 1 | 1 |
| Tafluprost/timolol maleate/Tapcom + Brinzolamide/Azopt | 1 | 1 |
| Tafluprost/timolol maleate/Tapcom + Ripasudil/Glanatec + Brinzolamide/Azopt | 1 | 1 |
| Travoprost/timolol/DuoTrav | 1 | 2 |
| Travoprost/timolol/DuoTrav + Brinzolamide/Azopt | 1 | 1 |
