# Supplementary material for: Conjunctival structure of glaucomatous eyes treated with anti-glaucoma eye drops: a cross-sectional study using anterior segment optical coherence tomography
Source: BMC Ophthalmol. 2020 Jun 19;20:244. doi: 10.1186/s12886-020-01518-6 (PMC7304144; doi:10.1186/s12886-020-01518-6)
Supplement: Supplementary file 3 — Additional file 3: Supplemental file 3. The univariate and general linear mixed model (GLMM) analyses on the conjunctival epithelium/conjunctival stroma preservation rate. a. The effects of background characteristics. b. The effects of anti-glaucoma eye drops. [file 12886_2020_1518_MOESM3_ESM.docx]

**Supplemental file 3. The univariate and general linear mixed model (GLMM) analyses on the conjunctival epithelium/conjunctival stroma preservation rate**

Supplemental file 3a. The effects of background characteristics

|  |  | Univariate analysis | | | |  | Multiple analysis 1 | | | |
| --- | --- | --- | --- | --- | --- | --- | --- | --- | --- | --- |
|  |  | B | SE | β | p-value |  | B | SE | β | p-value |
| Sex (male vs. female) |  | -0.003 | 0.003 | −0.050 | 0.28 |  | -0.004 | 0.003 | −0.057 | 0.234 |
| Age (years) |  | 0.19e-3 | 0.11e-3 | −0.11 | **0.033** |  | 0.19e-3 | 0.11e-3 | −0.11 | **0.038** |
| Number of anti-glaucoma eye drops |  | -0.001 | 0.001 | −0.045 | 0.326 |  | -0.002 | 0.001 | −0.089 | 0.132 |
| Duration of administration (months) |  | 0.30e-4 | 0.10e-3 | 0.015 | 0.768 |  | 0.15e-3 | 0.12e-3 | 0.077 | 0.227 |

Supplemental file 3b. The effects of anti-glaucoma eye drops

|  |  | Univariate analysis | | | |  | Multiple analysis 2 | | | |  | Multiple analysis 3 | | | |
| --- | --- | --- | --- | --- | --- | --- | --- | --- | --- | --- | --- | --- | --- | --- | --- |
|  |  | B | SE | β | p-value |  | B | SE | β | p-value |  | B | SE | β | p-value |
| Prostaglandin analogs |  | -0.003 | 0.003 | −0.046 | 0.311 |  | -0.003 | 0.003 | –0.047 | 0.299 |  | n.e. |  |  |  |
| α2-receptor agonist |  | -0.006 | 0.004 | −0.065 | 0.160 |  | -0.006 | 0.004 | −0.064 | 0.161 |  | n.e. |  |  |  |
| Rho kinase inhibitor |  | 0.005 | 0.006 | 0.031 | 0.440 |  | 0.004 | 0.006 | 0.028 | 0.488 |  | n.e. |  |  |  |
| The fixed combination of β-blockers/CAIs |  | -0.002 | 0.004 | −0.025 | 0.564 |  | -0.002 | 0.004 | −0.024 | 0.579 |  | n.e. |  |  |  |
| The fixed combination of β-blockers/prostaglandin analogs |  | -0.006 | 0.005 | −0.052 | 0.303 |  | -0.006 | 0.005 | −0.050 | 0.313 |  | n.e. |  |  |  |
| β-blockers |  | 0.002 | 0.010 | 0.012 | 0.821 |  | 0.001 | 0.010 | 0.006 | 0.909 |  | n.e. |  |  |  |
| CAIs |  | -0.005 | 0.006 | −0.038 | 0.449 |  | -0.006 | 0.006 | −0.048 | 0.333 |  | n.e. |  |  |  |

Multiple analysis 1, multiple GLMM analysis with the preservation rate and with background characteristics; Multiple analysis 2, multiple GLMM analysis with the preservation rate and with the eye drops after adjusted for confounding factor (age).

CAI, carbonic anhydrase inhibitors SE, standard error; n.e., not entered (excluded variables in the forward selection method)
