# Supplementary material for: Conjunctival structure of glaucomatous eyes treated with anti-glaucoma eye drops: a cross-sectional study using anterior segment optical coherence tomography
Source: BMC Ophthalmol. 2020 Jun 19;20:244. doi: 10.1186/s12886-020-01518-6 (PMC7304144; doi:10.1186/s12886-020-01518-6)
Supplement: Supplementary file 4 — Additional file 4: Supplemental file 4. The univariate and general linear mixed model (GLMM) analyses on the conjunctival stroma/Tenon’s capsule preservation rate. a. The effects of background characteristics. b. The effects of anti-glaucoma eye drops. [file 12886_2020_1518_MOESM4_ESM.docx]

**Supplemental file 4. The univariate and general linear mixed model (GLMM) analyses on the conjunctival stroma/Tenon’s capsule preservation rate**

Supplemental file 4a. The effects of background characteristics

|  |  | Univariate analysis | | | | | Multiple analysis 1 | | | |
| --- | --- | --- | --- | --- | --- | --- | --- | --- | --- | --- |
|  |  | B | SE | Β | p-value |  | B | SE | Β | p-value |
| Sex (male vs. female) |  | -0.006 | 0.036 | −0.010 | 0.867 |  | -0.016 | 0.019 | −0.028 | 0.377 |
| Age (years) |  | -0.0002 | 0.0012 | -0.0077 | 0.874 |  | 0.0004 | 0.0007 | 0.015 | 0.593 |
| Number of anti-glaucoma eye drops |  | -0.153 | 0.0080 | −0.654 | **<0.001** |  | -0.110 | 0.009 | −0.454 | **<0.001** |
| Duration of administration (months) |  | -0.012 | 0.0008 | −0.660 | **<0.001** |  | -0.006 | 0.0008 | −0.350 | **<0.001** |

Supplemental file 4b. The effects of anti-glaucoma eye drops

|  |  | Univariate analysis | | | |  | Multiple analysis 2 | | | |  | Multiple analysis 3 | | | |
| --- | --- | --- | --- | --- | --- | --- | --- | --- | --- | --- | --- | --- | --- | --- | --- |
|  |  | B | SE | β | p-value |  | B | SE | β | p-value |  | B | SE | β | p-value |
| Prostaglandin analogs |  | -0.416 | 0.028 | −0.586 | **<0.001** |  | -0.123 | 0.035 | −0.174 | **0.001** |  | -0.130 | 0.037 | −0.183 | **<0.001** |
| α2-receptor agonist |  | -0.324 | 0.041 | −0.388 | **<0.001** |  | 0.119 | 0.037 | 0.142 | **0.001** |  | 0.087 | 0.037 | 0.103 | **0.021** |
| Rho kinase inhibitor |  | -0.231 | 0.067 | −0.150 | **0.001** |  | 0.010 | 0.047 | −0.007 | 0.824 |  | n.e. |  |  |  |
| The fixed combination of β-blockers/CAIs |  | -0.344 | 0.033 | −0.439 | **<0.001** |  | -0.037 | 0.032 | −0.047 | 0.251 |  | n.e. |  |  |  |
| The fixed combination of β-blockers/prostaglandin analogs |  | -0.356 | 0.061 | −0.337 | **<0.001** |  | -0.058 | 0.037 | −0.055 | 0.117 |  | -0.096 | 0.036 | −2.640 | **0.009** |
| β-blockers |  | -0.437 | 0.104 | −0.240 | **<0.001** |  | -0.070 | 0.067 | −0.039 | 0.295 |  | n.e. |  |  |  |
| CAIs |  | -0.285 | 0.069 | −0.243 | **0.009** |  | 0.097 | 0.041 | 0.083 | **0.019** |  | **n.e.** |  |  |  |

Multiple analysis 1, multiple GLMM analysis with the thickness of the preservation rate and with background characteristics; Multiple analysis 2, multiple GLMM analysis with the preservation rate and with the eye drops after adjusted for confounding factor (number of anti-glaucoma eye drops and duration of administration).; Multiple analysis 3, the stepwise method for variable selection.

CAI, carbonic anhydrase inhibitors; SE, standard error; n.e., not entered (excluded variables in the forward selection method)
