# Supplementary material for: Conjunctival structure of glaucomatous eyes treated with anti-glaucoma eye drops: a cross-sectional study using anterior segment optical coherence tomography
Source: BMC Ophthalmol. 2020 Jun 19;20:244. doi: 10.1186/s12886-020-01518-6 (PMC7304144; doi:10.1186/s12886-020-01518-6)
Supplement: Supplementary file 5 — Additional file 5: Supplemental file 5. The univariate and general linear mixed model (GLMM) analyses on the Tenon’s capsule/sclera preservation rate. a. The effects of background characteristics. b. The effects of anti-glaucoma eye drops. [file 12886_2020_1518_MOESM5_ESM.docx]

**Supplemental file 5. The univariate and general linear mixed model (GLMM) analyses on the Tenon’s capsule/sclera preservation rate**

Supplemental file 5a. The effects of background characteristics

|  |  | Univariate analysis | | | |  | Multiple analysis 1 | | | |
| --- | --- | --- | --- | --- | --- | --- | --- | --- | --- | --- |
|  |  | B | SE | β | p-value |  | B | SE | β | p-value |
| Sex (male vs. female) |  | -0.005 | 0.034 | −0.0083 | 0.886 |  | -0.016 | 0.018 | −0.027 | 0.384 |
| Age (years) |  | -0.0001 | 0.001 | -0.0036 | 0.941 |  | 0.0006 | 0.0007 | 0.027 | 0.354 |
| Number of anti-glaucoma eye drops |  | -0.141 | 0.008 | −0.624 | **<0.001** |  | -0.092 | 0.0089 | −0.41 | **<0.001** |
| Duration of administration (months) |  | -0.011 | 0.0007 | −0.653 | **<0.001** |  | -0.0065 | 0.0007 | −0.37 | **<0.001** |

Supplemental file 5b. The effects of anti-glaucoma eye drops

|  |  | Univariate analysis | | | |  | Multiple analysis 2 | | | |  | Multiple analysis 3 | | | |
| --- | --- | --- | --- | --- | --- | --- | --- | --- | --- | --- | --- | --- | --- | --- | --- |
|  |  | B | SE | β | p-value |  | B | SE | β | p-value |  | B | SE | β | p-value |
| Prostaglandin analogs |  | -0.388 | 0.026 | −0.569 | **<0.001** |  | -0.132 | 0.034 | −0.193 | **<0.001** |  | -0.130 | 0.035 | −0.191 | **<0.001** |
| α2-receptor agonist |  | -0.284 | 0.039 | −0.352 | **<0.001** |  | 0.149 | 0.035 | 0.186 | **<0.001** |  | 0.117 | 0.035 | 0.146 | **0.001** |
| Rho kinase inhibitor |  | -0.291 | 0.064 | −0.196 | **<0.001** |  | -0.067 | 0.046 | −0.045 | 0.147 |  | n.e. |  |  |  |
| The fixed combinations of β-blockers/CAIs |  | -0.329 | 0.032 | −0.435 | **<0.001** |  | -0.052 | 0.032 | −0.069 | **0.102** |  | **n.e.** |  |  |  |
| The fixed combinations of β-blockers/prostaglandin analogs |  | -0.326 | 0.058 | −0.321 | **<0.001** |  | -0.053 | 0.036 | −0.053 | 0.136 |  | -0.092 | 0.034 | −0.091 | **0.008** |
| β-blockers |  | -0.262 | 0.101 | −0.149 | **0.001** |  | -0.008 | 0.065 | −0.004 | 0.907 |  | n.e. |  |  |  |
| CAIs |  | -0.265 | 0.065 | −0.234 | **<0.001** |  | 0.079 | 0.041 | 0.070 | **0.052** |  | **n.e.** |  |  |  |

Multiple analysis 1, multiple GLMM analysis with the thickness of the preservation rate and with background characteristics; Multiple analysis 2, multiple GLMM analysis with the preservation rate and with the eye drops after adjusted for confounding factor (number of anti-glaucoma eye drops and duration of administration).; Multiple analysis 3, the stepwise method for variable selection.

CAI, carbonic anhydrase inhibitors; SE, standard error; n.e., not entered (excluded variables in the forward selection method)
